# HFTC: a hierarchical fungal taxonomic classification model for ITS sequences using low-dimensional embedding features

**DOI:** 10.3389/fgene.2025.1650244

**Published:** 2025-10-03

**Authors:** Jiawei Wang, Shaojie Qiao, Dongsheng Xiang, Yangcheng Liao, Chao Wang

**Affiliations:** ^1^ School of Software Engineering, Chengdu University of Information Technology, Chengdu, China; ^2^ Center for Genomic and Personalized Medicine, Guangxi key Laboratory for Genomic and Personalized Medicine, Guangxi Collaborative Innovation Center for Genomic and Personalized Medicine, University Engineering Research Center of Digital Medicine and Healthcare, Guangxi Medical University, Nanning, Guangxi, China

**Keywords:** fungal identification, ITS sequencing, hierarchical classification, Word2Vec embedding, random forests

## Abstract

**Introduction:**

Fungal identification through ITS sequencing is pivotal for biodiversity and ecological studies, yet existing methods often face challenges with high-dimensional features and inconsistent taxonomy predictions.

**Method:**

We proposed HFTC, a hierarchical fungal taxonomic classifier built upon a multi-level random forest (RF) architecture. Notably, HFTC incorporates a bidirectional k-mer strategy to capture contextual information from both sequence orientations. By leveraging Word2Vec embedding, it reduces feature dimensionality from 4^*k*^ to only 200, significantly improving computational efficiency while preserving rich sequence context.

**Result:**

Experimental results demonstrate that HFTC outperforms Mothur, RDP, Sintax, QIIME2, and CNN-Duong, achieving a Matthews correlation coefficient (MCC) of 95.31% despite uneven class distributions. Its overall accuracy (ACC) reaches 95.25%. At the species level, it attains a hierarchical accuracy (HA) of 95.10%, surpassing the best-performing deep learning baseline, CNN-Duong, by 3.2%. Moreover, HFTC exhibits the smallest discrepancy between ACC and HA (1.60%), in contrast to CNN-Duong, which shows the largest gap (35.00%), highlighting HFTC’s superior hierarchical consistency.

**Discussion:**

HFTC offers a scalable and accurate approach for fungal taxonomic classification. Its compact feature representation and hierarchical architecture make it particularly suitable for microbial diversity research. The source code and datasets are publicly accessible at https://github.com/wjjw0731/HFTC/tree/master.

## 1 Introduction

Fungi are indispensable to the Earth’s ecosystem balance and have a profound impact on human life (Shoemaker et al., 2017) for their essential roles in biodiversity conservation, organic matter decomposition, medicine and food production, and agricultural bio-control application (Lennon and Locey, 2020). Despite their ecological and biotechnological significances, fungi remain vastly underexplored. Recent estimates suggest that approximately 12 million fungal species may exist, yet only approximately 150,000 have been formally described (Hawksworth and Lücking, 2017), implying that over 99% of fungal diversity remains undocumented. Therefore, accurate and scalable species identification is essential to advance microbial diversity research and functional inference.

Traditional fungal identification approaches, based on morphology, anatomy, or sectional analysis (Raja et al., 2017), are impractical when morphological traits are absent or ambiguous. Although whole-genome sequencing provides the highest resolution for identification, it remains expensive, computationally intensive, and time-consuming (Gan et al., 2024; Saada et al., 2024). As a result, DNA metabarcoding has emerged as a widely adopted alternative for microbial community profiling. This approach targets a small, species-specific, and easily amplified genomic region (Raja et al., 2017; Ficetola et al., 2010). Several key rRNA gene regions, such as ITS, LSU, SSU, RPB2, and TEF, have been used for fungal species identification (Zhang et al., 2020; Schoch et al., 2012). Compared to LSU and SSU, which evolve slowly and lack resolution for closely related species, ITS provides superior species- and strain-level discrimination. Although slower-evolving than protein-coding markers such as RPB2 or TEF, ITS offers a balanced level of variability and conservation (Nilsson et al., 2019; Lindahl et al., 2013). Consequently, ITS has been designated as the universal DNA barcode for fungi (Bradshaw et al., 2023; Nilsson et al., 2008). To support ITS-based research, several databases, including UNITE (Kõljalg et al., 2020), Warcup (Deshpande et al., 2016), and BOLD (Ratnasingham and Hebert, 2007), have been developed. Among these, UNITE is comprehensive and frequently updated, containing nearly 10 million sequences grouped into 2.4 million species hypotheses (SHs) (Kõljalg et al., 2020). UNITE provides a valuable taxonomic database for taxonomists. However, it also presents challenges such as data noise, taxonomic imbalance, and ambiguous categories, which must be addressed for reliable classification.

Barcoding-based methods for microbiome classification are divided into alignment-based and alignment-free categories (Borozan et al., 2015). Alignment-based methods [e.g., BLAST (Altschul et al., 1990)] rely on pairwise sequence similarity searches, which are accurate but computationally intensive. In contrast, alignment-free methods offer speed and scalability and are increasingly adopted for high-throughput microbiome analysis (van Zyl et al., 2025; Zhou et al., 2019). These methods typically use k-mer frequency vectors (KFVs) for sequence representation (Jenike et al., 2024), leading to high-dimensional (4^*k*^), sparse representation, sensitivity to noise, and limited interpretability (Wichmann et al., 2023), which can hinder model performance and increase computational burden. Most existing models adopt a flat classification architecture, using a single model to predict all taxonomic ranks simultaneously. This structure lacks hierarchical awareness and often yields inconsistent predictions. For instance, a sample misclassified at a higher level (e.g., phylum or class) may still appear correct at lower levels (e.g., genus or species), which is biologically invalid. Despite this, accuracy at each level is typically reported in isolation, without accounting for upstream errors. For instance, if one sequence has the correct phylum but incorrect class and another has the wrong phylum but correct class, one of them will always be counted as correct in single-rank evaluations (either at the phylum or class level), although both are taxonomically inconsistent. This can inflate the perceived performance and obscure the true reliability of the model in practical taxonomic applications.

In this study, we address these challenges by proposing HFTC, a hierarchical fungal taxonomic classifier based on ITS sequences. First, to mitigate the effects of data imbalance and noise, we rigorously curated the UNITE database to construct a high-quality ITS dataset. Second, to reduce dimensionality and improve contextual representation, we adopted a bi-directional k-mer (Bi-kmer) strategy to capture richer sequence context information and applied Word2Vec embedding (Wichmann et al., 2023; Wang et al., 2020; Neelima and Mehrotra, 2023; Asim et al., 2020) to compress the feature space from 4^*k*^ to only 200 dimensions. Third, we developed a multi-level random forest (RF) architecture to ensure taxonomic consistency. Together, these efforts enable more accurate, efficient, and consistent classification of fungal species. Experimental results demonstrate that HFTC significantly outperforms baseline approaches in both feature dimensions and hierarchical consistency.

## 2 Materials and methods

**FIGURE 1:**
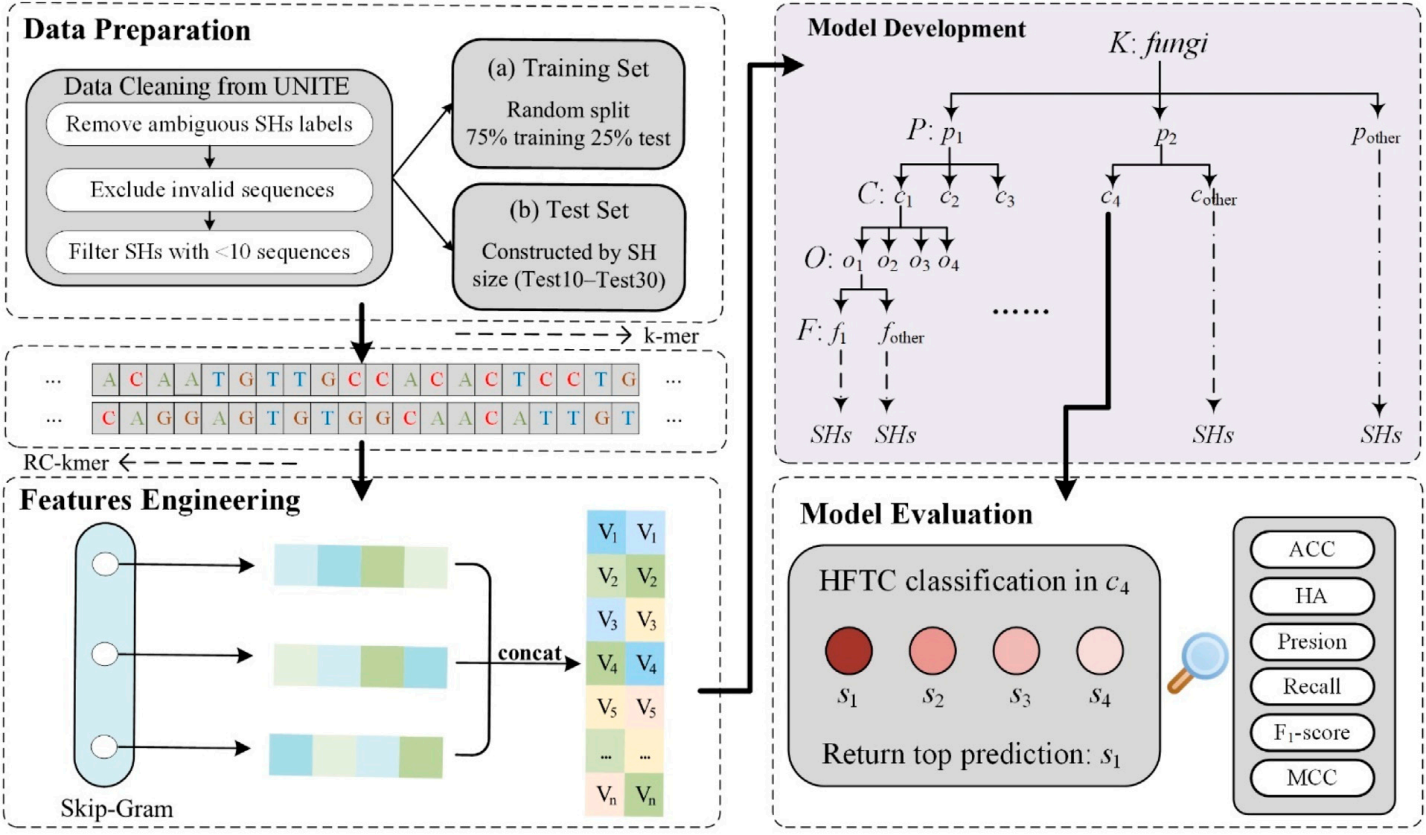
Schematic workflow of HFTC, a hierarchical fungal taxonomic classification framework. The pipeline consists of four main stages: data preparation, feature engineering, model development, and model evaluation.

Figure 1 illustrates the overall workflow of HFTC, which comprises four main stages—from raw ITS sequence processing to final taxonomic prediction—to address key challenges in fungal classification.

### 2.1 Data extraction and preprocessing

The UNITE database focuses on the eukaryotic nuclear ribosomal ITS region, where sequences are clustered into SHs based on pairwise similarity thresholds of 0.5% (Kõljalg et al., 2013; Karsch-Mizrachi et al., 2018). For this study, we retrieved the full fungal ITS dataset from the UNITE dataset (v9.0) (UNITE Community, 2023), which integrates fungal sequences from both UNITE and INSD. The crude dataset includes 6,499,364 sequences.

To ensure data quality, we implemented a rigorous data preprocessing pipeline. First, 1,325,964 sequences labeled as unidentified or ambiguous (e.g., *incertae sedis*) and 66,707 erroneous sequences containing non-standard nucleotide bases were excluded (Nilsson et al., 2018). To further improve annotation reliability and reduce noise, 295,108 sequences from SHs with fewer than 10 representative sequences were excluded. These rare taxa typically lack sufficient intra-class variation to support stable training and are more susceptible to misannotation, potentially introducing bias. This filtering process represents a necessary trade-off between taxonomic inclusiveness and model reliability. Given the scale of this study—spanning over 25,000 fungal SHs—it constitutes a challenging large-scale multi-class classification task. The exclusion of underrepresented SHs has minimal impact on overall taxonomic coverage as the curated dataset already captures the major fungal lineages. Instead, this strategy significantly improves training efficiency and consistency without compromising fungal diversity.

Among the remaining SHs, the number of sequences varied widely, ranging from 10 to tens of thousands. To address this imbalance, we randomly sampled 10 sequences from each SH with more than 10 sequences. This step minimized sequence redundancy and ensured uniform representation across taxa. Since SHs in the UNITE database are clustered based on ITS sequence similarity and serve as proxies for species, this sampling strategy not only balances data distribution but also approximates species-level stratified sampling. It helps prevent model overfitting to overrepresented taxa, thereby promoting robustness and generalizability. As a result, the final training set comprised 251,630 sequences representing 25,163 fungal species. The data and associated metadata have been deposited in Zenodo in accordance with community metadata standards, available at https://zenodo.org/uploads/14826761.

To rigorously assess model performance, we constructed five independent test sets using only sequences that were completely absent from the training set, ensuring that no sequences overlap. In particular, we constructed five independent test sets (Test10–Test30) by randomly selecting 10–30 representative sequences per species, respectively. Dataset distributions are summarized in Table 1, and the most species-rich taxa at each taxonomic level are given in Supplementary Image S1.

**TABLE 1:** Taxonomic coverage information for training and testing datasets.

Dataset	Kingdom	Phylum	Class	Order	Family	Genus	Species	Total no.
Training	1	15	56	181	604	2,683	25,163	251,630
Test10	1	15	52	164	520	2,034	15,027	150,270
Test15	1	14	48	146	452	1,646	9,965	149,475
Test20	1	12	44	138	417	1,398	7,082	141,640
Test25	1	12	40	120	360	1,165	5,304	132,600
Test30	1	9	36	110	335	1,029	4,149	124,441

### 2.2 Sequence feature representation

In this work, we used Word2Vec to embed ITS sequences into dense numerical vectors. Each ITS sequence was regarded as a sentence, with k-mers serving as words whose contextual patterns capture taxonomic information. To construct a comprehensive sequence corpus, k-mers were extracted in both the forward and reverse directions (Marçais et al., 2024) using a sliding window of length *k* and stride length *L*. This bidirectional approach captures richer local sequence context and improves embedding robustness. For Word2Vec training, we adopted the skip-gram model instead of the Continuous Bag-of-Words (CBOW) model. Skip-gram performs better in capturing rare or infrequent k-mers by directly predicting surrounding context words from a given center word (TH et al., 2015). In contrast, CBOW averages the context to predict the center word, which tends to oversmooth representations and underperform on sparse biological sequences such as fungal ITS data (Chiu et al., 2016).

After training, each k-mer was mapped to an *N*-dimensional embedding vector. To represent a full ITS sequence, we computed the average of all embedded k-mer vectors from each direction separately and then concatenated them to obtain a final 2*N*-dimensional sequence-level vector.

### 2.3 Construction of HFTC

To address the inconsistency of classification results across taxonomic levels, we proposed HFTC, a novel model that aligned predictions with phylogenetic relationships from phylum to species (Ji et al., 2023). Unlike conventional flat models, which predict all taxonomic ranks simultaneously and may yield spuriously high accuracy, HFTC decomposes the task into sequential subtasks, each handled by an independently trained sub-classifier. By integrating predictions across levels, HFTC reduces overall complexity and ensures taxonomic consistency (Zhang and Zhou, 2013).

To further support this hierarchical design, we adopted random forests (Breiman, 2001) as the base classifiers for each level. Compared with more complex models such as neural networks (NNs), RFs are more robust to class imbalance, require fewer computational resources, and are less sensitive to hyperparameter tuning (Fernández-Delgado et al., 2014). Moreover, the hierarchical tree-like structure of HFTC naturally aligns with the modular design, whereas NNs struggle with vanishing gradients and poor generalization in long-tailed settings (Zhang et al., 2023; Le Guillarme and Thuiller, 2022). Additionally, many taxonomic groups contain very few sequences, making it infeasible to train dedicated classifiers. To address this, taxa with fewer than 1,000 sequences were grouped into an “Other” category. Due to the strong multi-class capability of RFs, a single classifier could then be trained from this node to directly predict species-level labels, despite the diversity and imbalance within the group. This strategy reduces the number of required sub-classifiers and streamlines the classification pipeline. Table 2 summarizes the recursive construction of HFTC across taxonomic levels.

**TABLE 2:** Training procedure for the Hierarchical Fungal Taxonomic Classifier (HFTC).

Algorithm 1 training HFTC
**Input** Sequences for ITS_10 Dataset Extracted sequence features **Output** Species-level taxonomics for fungal ITS sequences 1: Start taxonomic classification at the Kingdom level 2: Construct a sub-classifier to predict the Phylum level 3: **if** any Phylum contains fewer than 1,000 species **then** 4: Group it as “Other Phyla” for direct species-level classification 5: **end if** 6: Continue to the next taxonomic level 7: **while** taxonomic levels remain to be classified **do** 8: Construct a sub-classifier to predict the next lower taxonomic level 9: **if** the lower-level taxonomic group contains fewer than 1,000 species **then** 10: Group it as “Other” for direct species-level classification 11: **end if** 12: Continue descending through the taxonomic hierarchy 13: **end while** 14: Perform direct species-level classification on all remaining taxonomic groups 15. **return** Species-level taxonomics for fungal ITS sequences

### 2.4 Model evaluation metrics

We adopt five metrics as foundational indicators to evaluate model’s performance: accuracy (ACC), recall, F_1_-score (F_1_), precision, and Matthews correlation coefficient (MCC) (GMJP and o, 2023; Boughorbel et al., 2017). They were calculated using Equations 1–5:

$$\text{ACC}=\frac{\text{TP}+\text{TN}}{\text{TP}+\text{TN}+\text{FP}+\text{FN}},$$ (1)

$$\text{Recall}=\frac{\text{TP}}{\text{TP}+\text{FN}},$$ (2)

$$\text{Precision}=\frac{\text{TP}}{\text{TP}+\text{FP}},$$ (3)

$$F_{1}=2\cdot \frac{\text{TP}}{2\cdot \text{TP}+\text{FP}+\text{FN}},$$ (4)

$$\text{MCC}=\frac{\text{TP}\times \text{TN}-\text{FP}\times \text{FN}}{\sqrt{\left(\text{TP}+\text{FP}\right)\left(\text{TP}+\text{FN}\right)\left(\text{TN}+\text{FP}\right)\left(\text{TN}+\text{FN}\right)}},$$ (5)

where TP represents true positive, FP represents false positive, TN represents true negative, and FN represents false negative.

Conventional metrics in taxonomic classification assess performance at individual levels but may overlook inconsistencies across the hierarchy. To address this limitation, we adopted the hierarchical accuracy (HA) (Tieppo et al., 2022) metric. HA counts a sample as a true positive only if all its hierarchical predictions are correct, quantifying fully correctly classified samples across the taxonomic path, as presented in Equation 6:

$$\text{HA}=\frac{{\text{TP}}^{*}}{\text{TP}+\text{TN}+\text{FP}+\text{FN}},$$ (6)

where TP^*^ denotes the true positives on the complete taxonomic classification path.

## 3 Result and discussion

### 3.1 Division of sub-classifiers in HFTC

HFTC is hierarchically constructed based on fungal phylogenetic taxonomy. In particular, an RF classifier first assigns sequences to phyla at the kingdom level. Phyla with fewer than 1,000 SHs are grouped into “Other Phyla” for direct species-level classification. For the major phyla, further hierarchical classification is performed. In this study, *Basidiomycota* and *Ascomycota* are the two most abundant phyla; in this study, *Basidiomycota* is divided into four specific classes and one “Other Class” category, while *Ascomycota* is divided into *Agaricomycetes* and “Other Class.” At the order level, 11 orders, except *Agaricomycetes*, were trained at the species level due to a significant reduction in species per order. For *Agaricomycetes*, RF classifiers are constructed at five order and four family levels. As a result, we constructed a total of 21 RF-based sub-classifiers. Considering the substantial variation in task complexity among sub-classifiers—ranging from dozens to tens of thousands of categories—establishing a unified confidence threshold becomes challenging. Given that HFTC’s hierarchical architecture effectively mitigates error propagation, we opted against implementing a confidence-based early stopping mechanism. Instead, our approach leverages the highest-confidence predictions from each RF-based sub-classifier as the final output, ensuring robust taxonomic assignments while maintaining computational efficiency. Nonetheless, users are allowed to apply confidence thresholds depending flexibly on task-specific requirements.

### 3.2 Strategy and optimization for feature embedding

#### 3.2.1 Evaluating the performance of various *k*-values in HFTC

**FIGURE 2:**
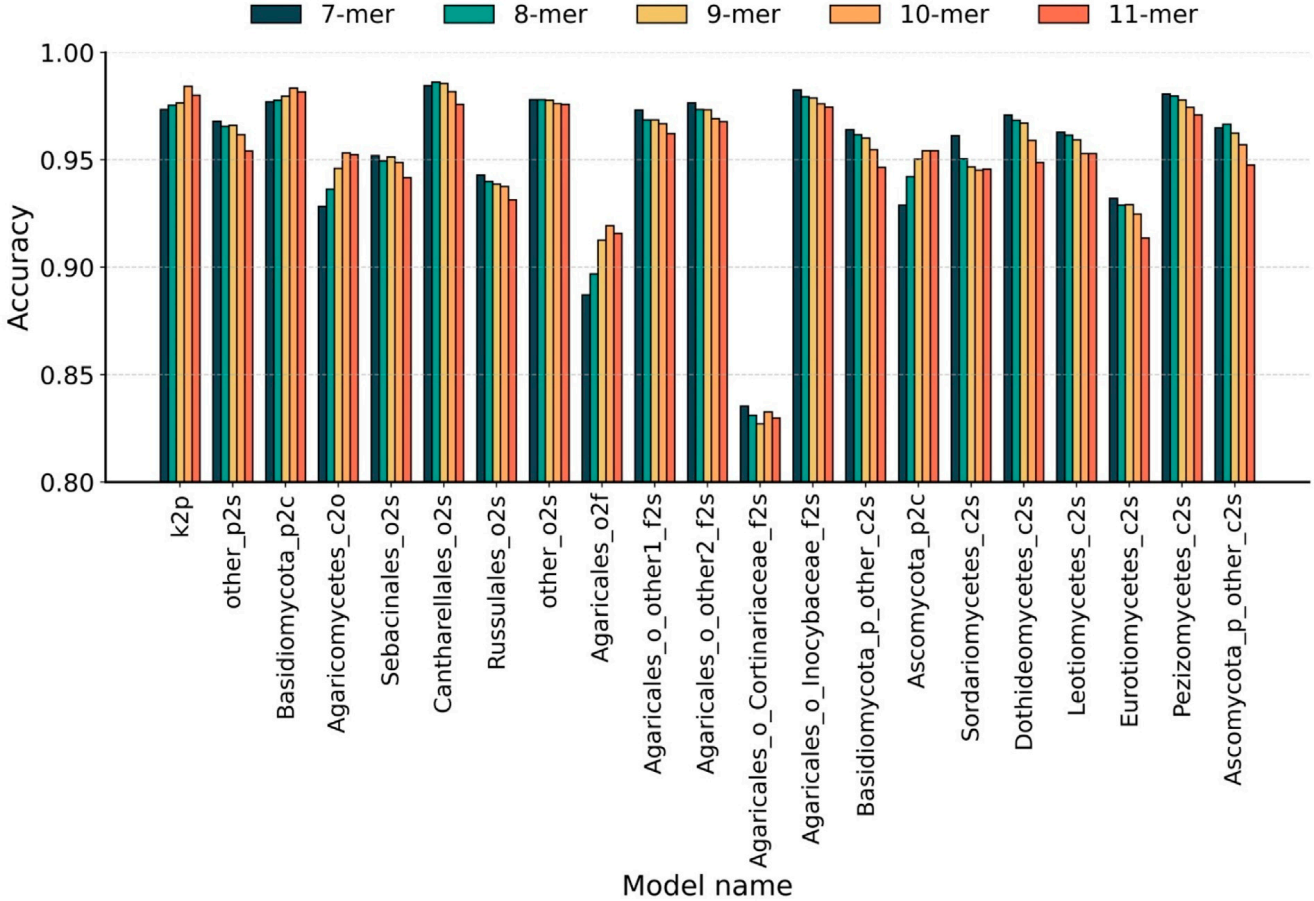
Taxonomic-level optimization of Bi-kmer length. Classification accuracy of 21 sub-classifiers evaluated across Bi-kmer lengths (*k* = 7–11) on the Test10 dataset. Taxonomic levels are abbreviated as follows: k, kingdom; p, phylum; c, class; o, order; f, family; s, species.

To optimize feature representation across the 21 derived hierarchical sub-classifiers, we systematically evaluated the optimal Bi-kmer length *k* for each taxonomic level by five-fold cross-validation. Initially, all sequences were represented as Bi-kmers and then embedded into 200-dimensional numerical vectors using Word2Vec. As the existing methods typically select *k*-values between 7 and 10 (Schloss et al., 2009; Wang et al., 2007; Edgar, 2016; Liimata et al., 2022; Vu et al., 2020), we evaluated the *k*-values from 7 to 11 to select the optimal feature representation at each taxonomic level. The accuracy achieved by sub-classifiers using individual *k*-values is presented in Figure 2. The hierarchical classification paths and optimal *k*-values of each sub-classifiers are detailed in Supplementary Table S1.

The results indicate that classifiers across different taxonomic levels exhibit an opposite trend in their optimal k-mer lengths. At higher taxonomic levels (phylum to family), classification accuracy generally peaked at *k* = 10. In particular, for phylum-level classification, the sub-classifier achieved the highest accuracy of 98.42% at *k* = 10, followed by 98.01% at *k* = 11, representing a 1.08% improvement over 97.34% at *k* = 7. For two representative class-level classifiers, the highest accuracies were observed at *k* = 10 for 98.34% (vs. 98.16% at *k* = 11) and *k* = 11 for 95.43% (vs. 95.42% at *k* = 10). For order- and family-level classifications, *k* = 10 again yielded the best accuracy—95.32% and 91.93%, respectively—surpassing all other tested *k*-values. In contrast, species-level classification exhibited an opposite trend, favoring shorter k-mers. Among the 16 species-level sub-classifiers, 13 achieved peak accuracy at *k* = 7. Two exceptional cases achieved optimal performance with *k* = 8 for 98.62% (vs. 98.45% at *k* = 7) and 96.66% (vs. 96.48% at *k* = 7). One sub-classifier showed equal performance at *k* = 7 and *k* = 8 for 97.80%. As stated above, we identify *k* = 7 and *k* = 10 as the optimal single *k*-value of k-mer for species-level and higher-level taxonomic classification tasks, respectively.

**FIGURE 3:**
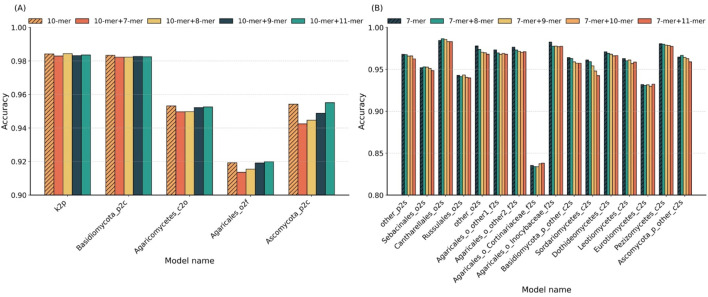
Comparison of single vs. hybrid k-mer strategies across taxonomic levels. **(A)** Accuracy of single *k* = 10 and its hybrid combinations (10 + 7, 10 + 8, 10 + 9, and 10 + 11) in five higher-level sub-classifiers. **(B)** Accuracy of single *k* = 7 and its hybrid combinations (7 + 8, 7 + 9, 7 + 10, and 7 + 11) in 16 species-level sub-classifiers.

To systematically evaluate the impact of k-mer combinations on classification performance across different taxonomic levels, we compared the optimal single *k*-values with their adjacent k-mer combinations. Figure 3 presents the accuracy results of five classifiers at higher levels in panel A and sixteen sub-classifiers at the species level in panel B.

In higher-level classification (Figure 3A), using *k* = 10 achieved the highest accuracy in two out of five sub-classifiers. In the remaining three cases, *k* = 10 ranked second, with accuracy reductions of less than 1% compared to the best hybrid combinations. This indicates that the single *k* = 10 setting is sufficient for robust performance at broader taxonomic ranks. At the species level (Figure 3B), 10 of the 16 sub-classifiers yielded peak accuracy with single *k* = 7; three cases reached optimal accuracy with a combination of *k* = 7 and *k* = 8; one with the *k* = 7 and *k* = 9 combination; and two with the *k* = 7 and *k* = 11 combination. These results suggest that hybrid k-mer features do not offer a significant advantage over well-chosen single *k*-values.

Overall, the findings demonstrate that adopting the optimal single *k*-value (*k* = 10 for higher levels, *k* = 7 for species level) provides a favorable trade-off between accuracy and model simplicity, without the need for additional complexity introduced by combining multiple k-mer lengths.

#### 3.2.2 Biological and statistical rationale for k-mer selection

Biologically, sequences within the same genus often share highly similar overall structures, with distinguishing signals typically confined to subtle local variations such as point mutations and short insertions or deletions (indels) (Mahadani and Ghosh, 2014). Therefore, species-level classification demands sensitivity to fine-grained, localized sequence differences. Shorter k-mers (e.g., *k* = 7 in this study) are better suited to capturing these microvariations, particularly within the hypervariable regions of the ITS sequence, which are the primary source of discriminatory information among closely related fungal taxa (Liu et al., 2025). Statistically, shorter k-mers also increase the overlap between local motifs, enhancing the resolution of small-scale mutations. This dense representation improves the model’s ability to differentiate among species based on minimal but biologically meaningful sequence differences. In contrast, higher-level classifications (e.g., phylum or class) involve greater evolutionary divergence, which manifests as broader conserved motifs or structural variations (Tedersoo et al., 2018). Statistically, longer k-mers yield sparser but more distinctive representations, reducing feature redundancy and increasing theoretical entropy across the k-mer space (Wang et al., 2018). This enhances inter-class separability and improves the robustness and accuracy of classification at broader taxonomic ranks.

Experiments highlight the advantage of a hierarchical feature design, where shorter k-mers are better suited for capturing fine-grained sequence variations at the species level, and longer k-mers provide improved resolution for distinguishing broader taxonomic groupings. The ability of HFTC to adaptively select k-mer lengths according to taxonomic level is a key factor underlying its robust and accurate performance across the entire fungal taxonomic hierarchy.

#### 3.2.3 Evaluation of sequence features embedding using Word2Vec

After determining the optimal *k*-values for each taxonomic level, we further evaluated the accuracy of Word2Vec embeddings by comparing them to traditional KFVs and applied both methods to five representative class-to-species sub-classifiers within the phylum *Ascomycota*.

**FIGURE 4:**
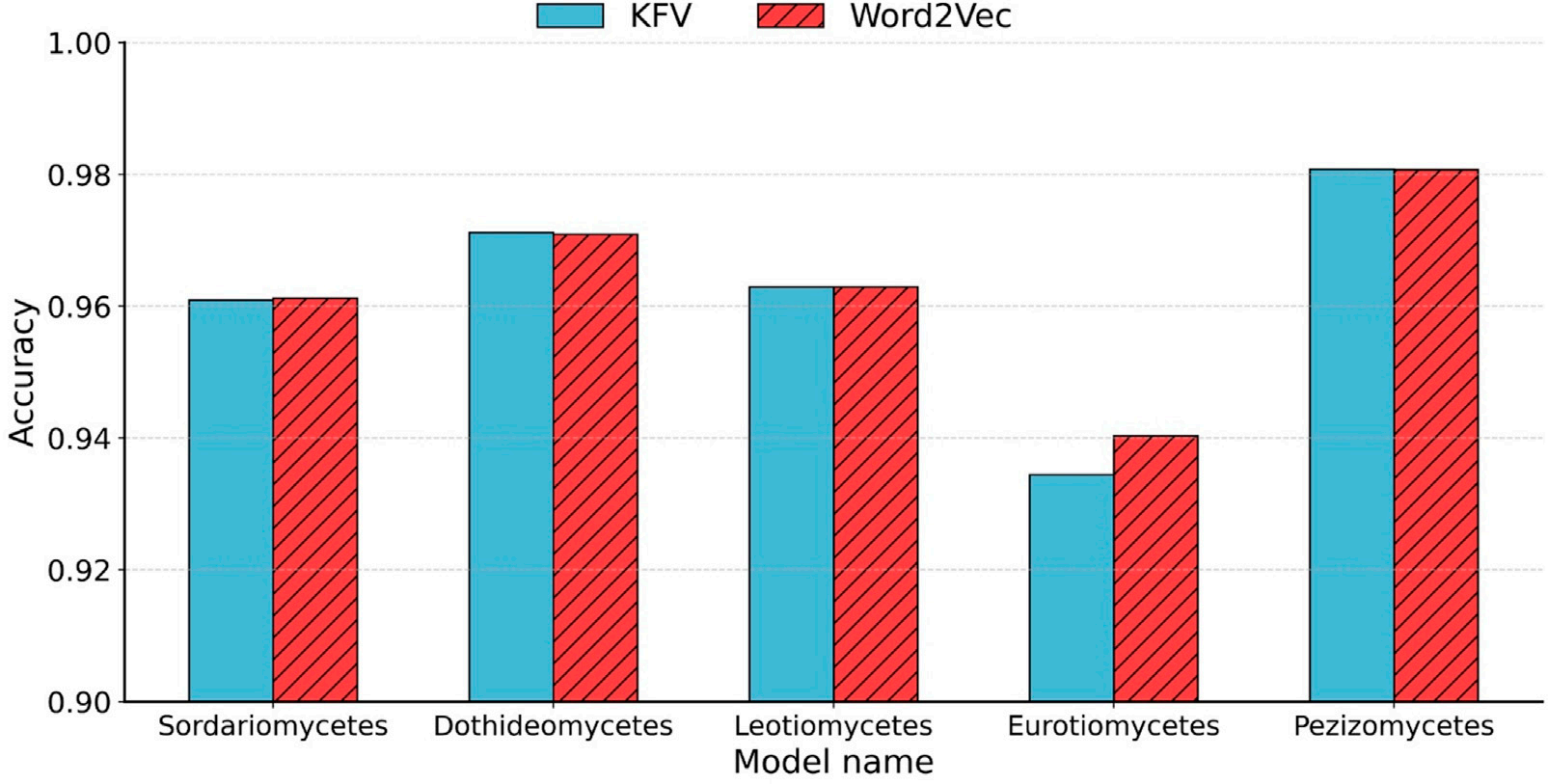
Accuracy comparison between KFVs and Word2Vec embedding. Accuracy of the five class-to-species sub-classifiers in *Ascomycota* using KFVs and Word2Vec embedding.

As shown in Figure 4, our method approach achieved an accuracy that was comparable to that of the KFV method. Traditional KFV methods produce extremely high-dimensional and sparse feature spaces (4^*k*^), causing memory bottlenecks during model training. In contrast, Word2Vec generates compact, dense representations by learning distributed vector embeddings for k-mers based on their contextual co-occurrence patterns. Importantly, our method reduced the feature dimensionality from 4^7^ (e.g., 16,384 for *k* = 7) to only 200 by applying average pooling over the Word2Vec-learned vectors of each Bi-kmer in the sequence, highlighting the embedding model’s ability to preserve relevant biological information with significantly fewer features.

Although averaging simplifies computation and mitigates noise from sequence length variation, it has limitations. In particular, it discards positional information, potentially overlooking structural motifs or taxonomically informative subsequences. Nonetheless, this embedding strategy significantly reduces feature dimensionality, mitigates sparsity, and captures essential compositional and contextual information for downstream classification tasks.

### 3.3 Model performance evaluation

#### 3.3.1 Hierarchical sub-classifier validation

The 21 sub-classifiers comprising the HFTC model were systematically evaluated through five-fold cross-validation in both the training and test datasets (Test_10). Three critical aspects are evaluated in Table 3(left: training set; right: test set): the number of taxonomic tasks covered (Shoemaker et al., 2017), the number of sequences (Lennon and Locey, 2020), and classification accuracy metrics (Hawksworth and Lücking, 2017).

**TABLE 3:** Information of sub-classifiers in the training and test datasets.

Model name	No. of taxa	No. of sequences	Accuracy
Fungi2p	15/15	251,630/150,270	0.9983/0.9998
other_p2s	1,312/757	13,111/7,564	0.9679/0.9533
Ascomycota_p2c	21/19	106,760/60,984	0.9542/0.9973
Sordariomycetes_c2s	2,868/1,620	28,465/16,041	0.9612/0.9562
Dothideomycetes_c2s	1,978/1,165	19,655/11,578	0.9709/0.9656
Leotiomycetes_c2s	1,294/801	12,816/7,968	0.9629/0.9610
Eurotiomycetes_c2s	2,023/1,173	20,016/11,629	0.9403/0.9236
Pezizomycetes_c2s	1,148/763	11,443/7,621	0.9807/0.9814
Ascomycota_p_other_c2s	1,444/616	14,365/6,174	0.9648/0.9639
Basidiomycota_p2c	14/13	131,759/81,722	0.9861/0.9983
Agaricomycetes_c2o	21/19	124,875/77,821	0.9532/0.9958
Agaricales_o2f	41/39	65,887/39,810	0.9193/0.9879
Cortinariaceae_f2s	1,175/679	11,733/6,772	0.8354/0.8071
Inocybaceae_f2s	1,377/982	13,761/9,820	0.9826/0.9778
Agaricales_o_other1_f2s	2,239/1,330	22,267/13,259	0.9778/0.9596
Agaricales_o_other2_f2s	1,835/2,989	18,126/29,851	0.9765/0.9742
Russulales_o2s	1,746/1,214	17,402/12,100	0.9429/0.9376
Sebacinales_o2s	1,030/634	10,300/6,340	0.9520/0.9395
Cantharellales_o2s	983/628	9,784/6,220	0.9845/0.9848
Agaricomycetes_c_other_o2s	2,164/1,344	21,502/13,351	0.9780/0.9648
Basidiomycota_p_other_c2s	689/392	6,884/3,901	0.9640/0.9693

Notably, the sub-classifiers maintained consistently high accuracy across all hierarchical taxonomic levels, with 17/21 achieving >95% classification accuracy (peak performance: *Fungi2p* reached 99.83% and 99.98% in training and test sets, respectively). This robust performance demonstrates the model’s dual capability in handling both coarse-grained (phylum-level) and fine-grained (species-level) taxonomic assignments. These results were all validated through 10-fold cross-validation experiments, with most of the standard deviations not exceeding 0.01, indicating stable model performance. The detailed standard deviations and 95% confidence intervals for the accuracy of each of the 21 sub-classifiers are also provided in Supplementary Table S1. Moreover, the minimal differences in accuracy between the training and test datasets highlight a strong generalization capability, indicating that the sub-classifiers are not overfitting and can reliably classify previously unseen data with high precision. However, the Cortinariaceae_f2s classifier for classifying species within the *Cortinariaceae* family of the order *Agaricales* performed poorly, with an accuracy of only 83.54% and 80.71% in training and test sets, respectively. In contrast, the classifier Inocybaceae_f2s for another family within *Agaricales* achieved an accuracy of 98.26%.

**FIGURE 5:**
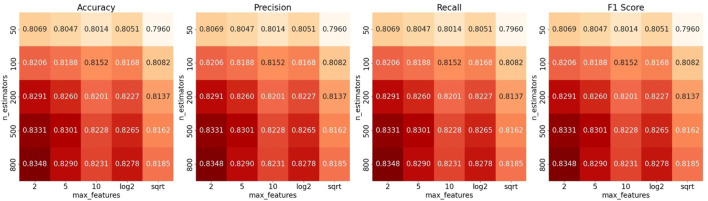
Hyperparameter optimization for Cortinariaceae_f2s via GridSearch. Accuracy, F_1_-score, precision, and recall values of the Cortinariaceae_f2s model constructed using different numbers of features and estimators.

To improve this model, we applied GridSearch on the training dataset to find the best performance combination. We tuned two hyperparameters: the number of features considered at each split (max_features) was tuned using a combination of fixed values and dynamic strategies commonly used in tree-based models, specifically {2, 5, 10, ‘log2’, ‘sqrt’} (Shoemaker et al., 2017). The dynamic options automatically select the number of features by taking either the base-2 logarithm or the square root of the total number of input features; the number of trees in the forest can be chosen from {50, 100, 200, 500, 800} (Lennon and Locey, 2020). Figure 5 shows heatmaps of ACC, recall, F_1_, and precision across the parameter combinations.

**FIGURE 6:**
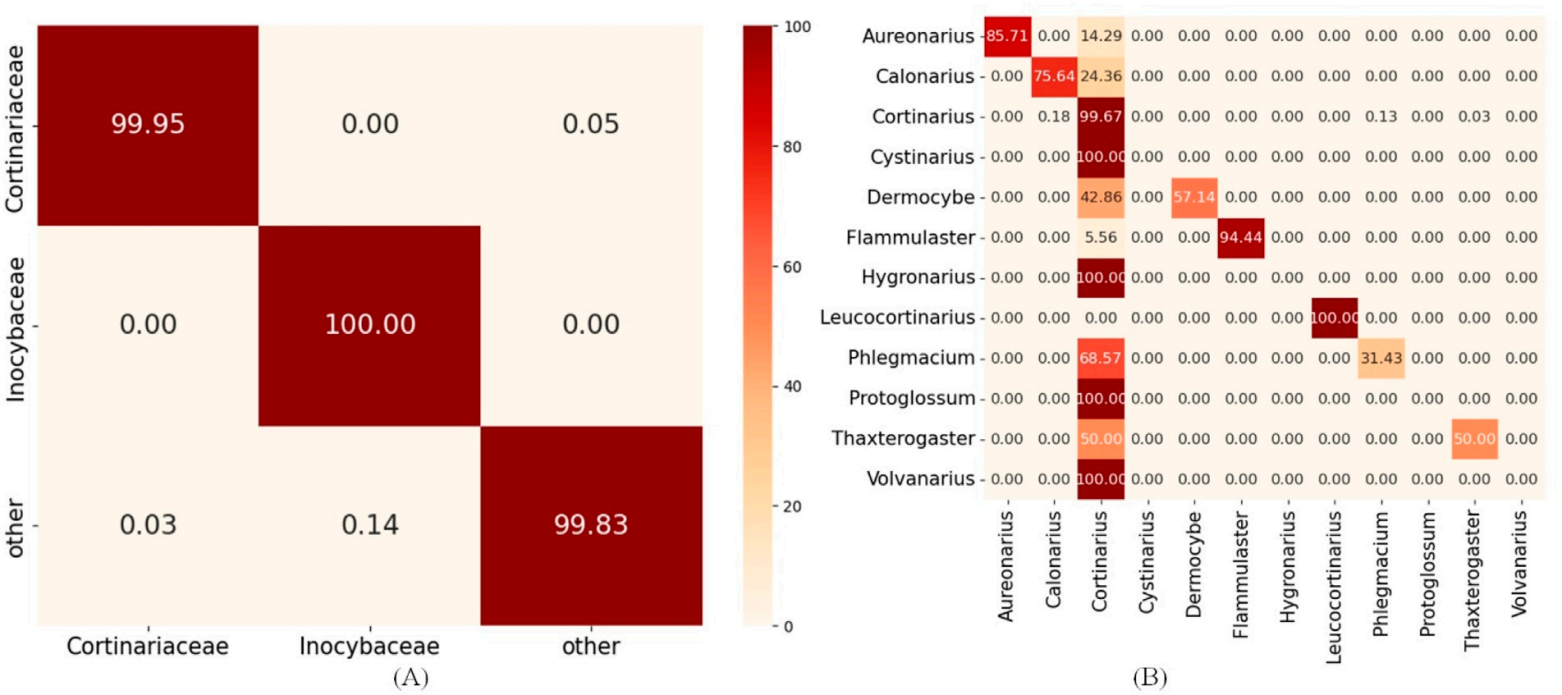
Hierarchical error propagation analysis in HFTC’s taxonomic classification pipeline. **(A)** Heatmaps of upstream tasks: Agaricales_o2f model and **(B)** heatmaps of downstream tasks: Cortinariaceae_f2g model.

When the two hyperparameters approached values of 2 and 800, the model achieved its highest accuracy of 0.8348. However, the model’s performance remained suboptimal despite extensive tuning via GridSearch. This indicates that the limited performance of the Cortinariaceae_f2s model is unlikely to be attributable to hyperparameter settings. Default RF parameters proved satisfactory results for most HFTC sub-classifiers; furthermore, tuning was avoided to prevent unnecessary complexity. To explore other potential causes, we examined the upstream and downstream tasks of the Agaricales_o2f and Cortinariaceae_f2g models, with the corresponding classification heatmaps presented in Figure 6.

The heatmaps reveal that the upstream classifier for the Agaricales order achieves nearly perfect accuracy in assigning sequences to the *Cortinariaceae* family for 99.95%, indicating that most errors in Cortinariaceae_f2s are not caused by upstream misclassification. Within the *Cortinariaceae* family, notable genus-level misclassifications occur, with many genera—particularly *Cystinarius*, *Hygronarius*, *Protoglossum*, and *Volvanarius*—erroneously predicted as *Cortinarius*. This bias likely stems from the dominance of Cortinarius in the training data and the limited representation of other genera. Additionally, *Cystinarius*, *Hygronarius*, and *Volvanarius*, which only recently split from *Cortinarius* (Liimata et al., 2022), remain phylogenetically close, leading to similar ITS sequences and reduced resolution in k-mer-based embeddings. These factors elevated false positives and degraded the performance of the Cortinariaceae_f2s classifier. To facilitate detailed analysis of how misclassifications at higher levels (e.g., phylum) affect downstream accuracy, we generated heatmaps of classification results for key taxonomic levels during training, provided in Supplementary Image S2.

#### 3.3.2 Comprehensive validation of the HFTC

After validating the robust performance of individual sub-classifiers, we conducted a comprehensive evaluation of the integrated HFTC system using six metrics across five independent test sets. Table 4 summarizes the performance of HFTC in the Test10 dataset, while detailed results for the other test sets are provided in Supplementary Table S2.

**TABLE 4:** Prediction performance for the HFTC in Test10.

Level	ACC	HA	Recall	Precision	F_1_	MCC
Phylum	0.9997	0.9997	0.9997	0.9997	0.9997	0.9994
Class	0.9943	0.9943	0.9967	0.9943	0.9954	0.9920
Order	0.9747	0.9744	0.9956	0.9747	0.9845	0.9724
Family	0.9648	0.9637	0.9648	0.9648	0.9799	0.9646
Genus	0.9525	0.9510	0.9508	0.9525	0.9544	0.9531
Species	0.9525	0.9510	0.9508	0.9525	0.9544	0.9531

Because HFTC achieves direct species-level classification from the family level, bypassing the intermediate genus level, the evaluation metrics at both the genus and species levels remained consistent, each exceeding 95%. Similar performance trends were observed across the remaining four independent test sets, with species-level accuracies of 94.7%, 94.1%, 93.5%, and 93.3%, respectively. In addition, in addressing class imbalance, the MCC surpassed 99.9% at the phylum level, 99.2% at the class level, 97.2% at the order level, 96.5% at the family level, and remained as high as 95.3% at the genus and species levels. These results fully demonstrate that HFTC can maintain robust performance even in the context of highly imbalanced classification tasks involving over 25,000 species. The high consistency between MCC and ACC further validates the model’s strong adaptability to imbalanced datasets.

### 3.4 Comparison with existing predictors

Considering representativeness and availability, five methods, namely, Mothur (Schloss et al., 2009), RDP (Wang et al., 2007), Sintax (Edgar, 2016), QIIME2 (Liimata et al., 2022), and CNN-Duong (Vu et al., 2020), were selected for model performance comparison on an independent and identical test dataset Test10. Mothur, Sintax, RDP, and QIIME2 use traditional machine learning, and CNN-Duong uses neural networks. In the experiments, Mothur, RDP, and Sintax were all executed on an AMD Ryzen Threadripper PRO 5955WX 16-Core Processor, using their recommended default parameters: a k-mer size of 8 and a confidence threshold of 0.9 for Sintax. QIIME2 was evaluated under the same environment with a k-mer size of 7 and a confidence threshold of 0.7, as recommended. Similarly, CNN-Duong was executed on GeForce RTX 4090 and evaluated using its default recommended settings, including a k-mer size of 6 and a CNN architecture, comprising two Conv1D layers and two dense layers, with a total of 49.83 MB of parameters.

**FIGURE 7:**
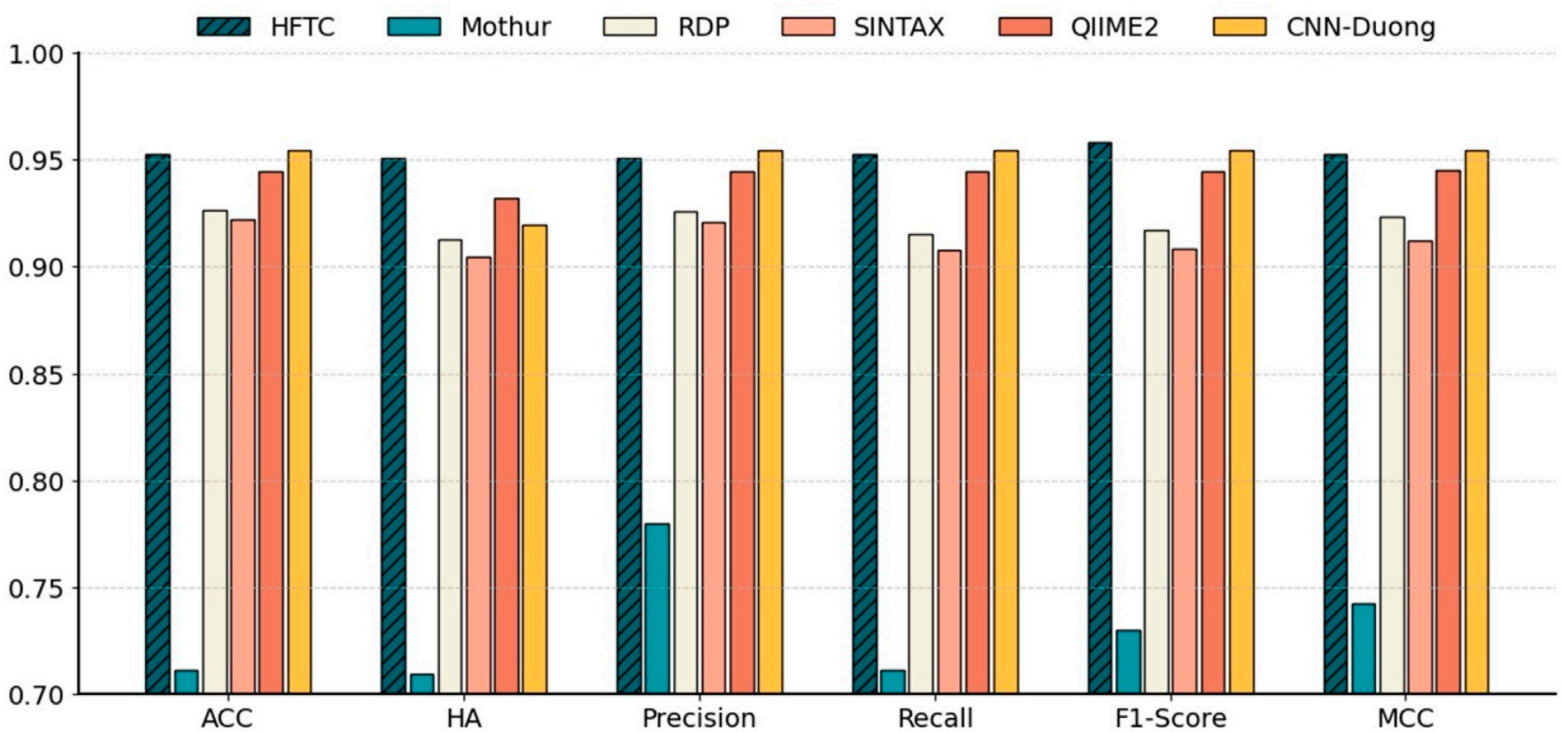
Comparison of six models at the species level in Test10. Benchmarking performance of HFTC against five state-of-the-art classifiers at the species level with six metrics: ACC, HA, precision, recall, F_1_-score, and MCC.

Figure 7 shows that HFTC achieved superior performance across all metrics, with an ACC of 95.25%, an HA of 95.09%, a precision of 95.08%, a recall of 95.25%, an F1-score of 95.80%, and an MCC of 95.31%. The CNN-Duong showed a slightly higher ACC of 95.43% but showed a marked decrease in HA to 91.90%, suggesting potential limitations in handling taxonomic hierarchies. QIIME2 ranked third overall, with both ACC and related metrics at approximately 94.44% and an HA of 93.21%. Notably, QIIME2, Sintax, and RDP are all Naïve Bayes-based methods; consistent with our earlier findings that *k* = 7 is optimal for species-level classification, QIIME2 (*k* = 7) outperformed RDP and Sintax (*k* = 8), which both achieved approximately 92% metrics. In addition, Mothur exhibited the poorest performance across all six evaluation metrics, showing an overall accuracy, hierarchical accuracy, precision, recall, F1 score, and MCC of 71.15%, 70.98%, 78.02%, 71.15%, 73.01%, and 74.28%, respectively.

In particular, Table 5 compares six models in terms of HA, species-level accuracy (ACC), feature vector size, and per-sequence inference time, which reflects the computational efficiency gained through dimensionality reduction. HFTC achieves the highest HA of 95.09% and a competitive ACC of 95.25%, only 0.18% lower than that of the best-performing CNN-Duong model. However, HFTC exhibits a substantial advantage in computational efficiency: its feature vector size is only 200, which is less than 0.3% of the 65,536-dimensional vectors used in traditional k-mer frequency-based models (Mothur, RDP, and Sintax), 1.2% of QIIME2’s 16,384, and only 4.8% of CNN-Duong’s 4,096. This low-dimensional embedding leads to significantly faster inference. HFTC achieves the fastest inference time among all six models, requiring only 0.37 milliseconds per sequence. It is 35% faster than the second-fastest model, Sintax (0.57 ms), and significantly outperforms the others—Mothur (0.92 ms), CNN-Duong (2.02 ms), RDP (12.25 ms), and QIIME2 (58.20 ms). Notably, HFTC achieves high speed without sacrificing accuracy, showing a good balance between efficiency and performance.

**TABLE 5:** In-depth comparison of six classifiers on the test10 dataset.

Method	Algorithm	ACC	HA	ACC-HA	Vector size	Inference time (ms)
HFTC	RF	0.9525	**0.9509**	**1.60%**	**200**	**0.37**
Mothur	KNN	0.7115	0.7098	1.73**%**	65,536	0.92
RDP	NB	0.9263	0.9125	13.73**%**	65,536	12.25
Sintax	NB	0.9221	0.9048	17.30**%**	65,536	0.57
QIIME	NB	0.9444	0.9321	12.36**%**	16,384	58.50
CNN-Duong	CNN	**0.9543**	0.9193	35.00**%**	4,096	2.02

Bold numbers indicate column-wise optimal values (highest ACC/HA, smallest ACC‐HA, vector size, and inference time).

Importantly, HFTC achieves the smallest gap between ACC and HA, with a difference of only 1.60%, indicating its strong ability to produce hierarchically consistent predictions. In contrast, although CNN-Duong attains a slightly higher accuracy of 95.43% at the species level, its HA decreases significantly to 91.93%, resulting in the largest ACC–HA gap of 35.00**%**. This performance gap reflects a fundamental limitation of CNN-based models with flat architectures, which do not explicitly capture taxonomic dependencies and are, therefore, more prone to inconsistent predictions across hierarchical levels. In contrast, HFTC uses a hierarchical progressive classifier selection mechanism that underpins its superior hierarchical consistency. Theoretically, this design also carries the potential risk of “higher-level misclassifications confining lower-level classifiers to incorrect branches.” However, HFTC mitigates this risk through two key features:

First, higher-level classifications in HFTC rely on highly discriminative inter-group features and undergo specialized training, minimizing misclassification rates and reducing the occurrence of incorrect branch guidance. Second, unlike flat models that use a single model to predict all taxonomic ranks simultaneously and yield inconsistent predictions, HFTC decomposes the task into sequential subtasks aligned with the biological taxonomic hierarchy. This inherent structural advantage explains why HFTC maintains such a narrow ACC–HA gap, outperforming flat architectures in preserving taxonomic integrity across all levels.

## 4 Conclusion

We presented HFTC, a bioinformatics tool for fungal species-level classification. To support a broad spectrum of fungal taxa, we constructed an ITS reference dataset encompassing over 25,000 species and 4.4 million sequences. HFTC incorporates three key innovations: a bi-kmer comprehensive feature extraction strategy that captures sequence context from both forward and reverse orientations (Shoemaker et al., 2017); Word2Vec embedding to compress high-dimensional KFV from 4^*k*^ into only 200-dimensional vectors, balancing computational efficiency with contextual information preservation (Lennon and Locey, 2020); and a hierarchical classification framework that ensures taxonomic consistency across levels (Hawksworth and Lücking, 2017). These innovations enable HFTC to achieve state-of-the-art performance at the species level, with 95.25% for ACC, 95.31% for MCC, and 95.10% for HA, while maintaining the smallest discrepancy between ACC and HA of 1.60**%**. HFTC shows excellent performance on fungal ITS data, and its architecture is generalizable to other barcoding systems such as 16S rRNA, with appropriate retraining and k-mer optimization.

Despite these strengths, HFTC has limitations that warrant future refinement. First, its performance is partially dependent on the quality of the reference database, highlighting the importance of accurate and comprehensive annotations such as those provided by UNITE. Second, although HFTC demonstrates scalability on large datasets, further optimization may be needed to efficiently handle ultra-large-scale sequencing data in future applications. Third, the k-mer pooling step in HFTC loses positional sequence information, which could limit resolution for closely related taxa with subtle structural variations. One promising direction is to leverage pre-trained language models (PLMs), such as DNABERT (Ji et al., 2021; Devlin et al., 2019), to complement HFTC’s current framework. These models could provide two key advantages: their ability to generate rich contextual embeddings from large-scale nucleotide corpora may help capture patterns from rare species that HFTC might overlook (Shoemaker et al., 2017), and their self-attention mechanisms and positional encoding capabilities could help recover the positional information lost during HFTC’s average pooling step, thereby enabling better use of local k-mer-based signals (Lennon and Locey, 2020). Although our study focused on the non-coding ITS region, the same principle could extend to coding markers such as RPB2 or TEF, which also contain taxonomically informative sequence motifs. Additionally, model compression techniques such as knowledge distillation could help reduce computational costs while maintaining accuracy. With these advancements, HFTC could further solidify its role as a cornerstone for scalable and accurate taxonomic identification in microbial research. All source code, datasets, and instructions for the experiments are publicly available at https://github.com/wjjw0731/HFTC/tree/master. The repository is documented and fully reproducible.

## Data Availability

The datasets presented in this study can be found in online repositories. The names of the repository/repositories and accession number(s) can be found at: Zenodo home: https://zenodo.org/uploads/14826761 DOI:10.5281/zenodo.14826761.
