# Supplementary figures and images for: HFTC: a hierarchical fungal taxonomic classification model for ITS sequences using low-dimensional embedding features
Source: Front Genet. 2025 Oct 3;16:1650244. doi: 10.3389/fgene.2025.1650244 (PMC12531816; doi:10.3389/fgene.2025.1650244)

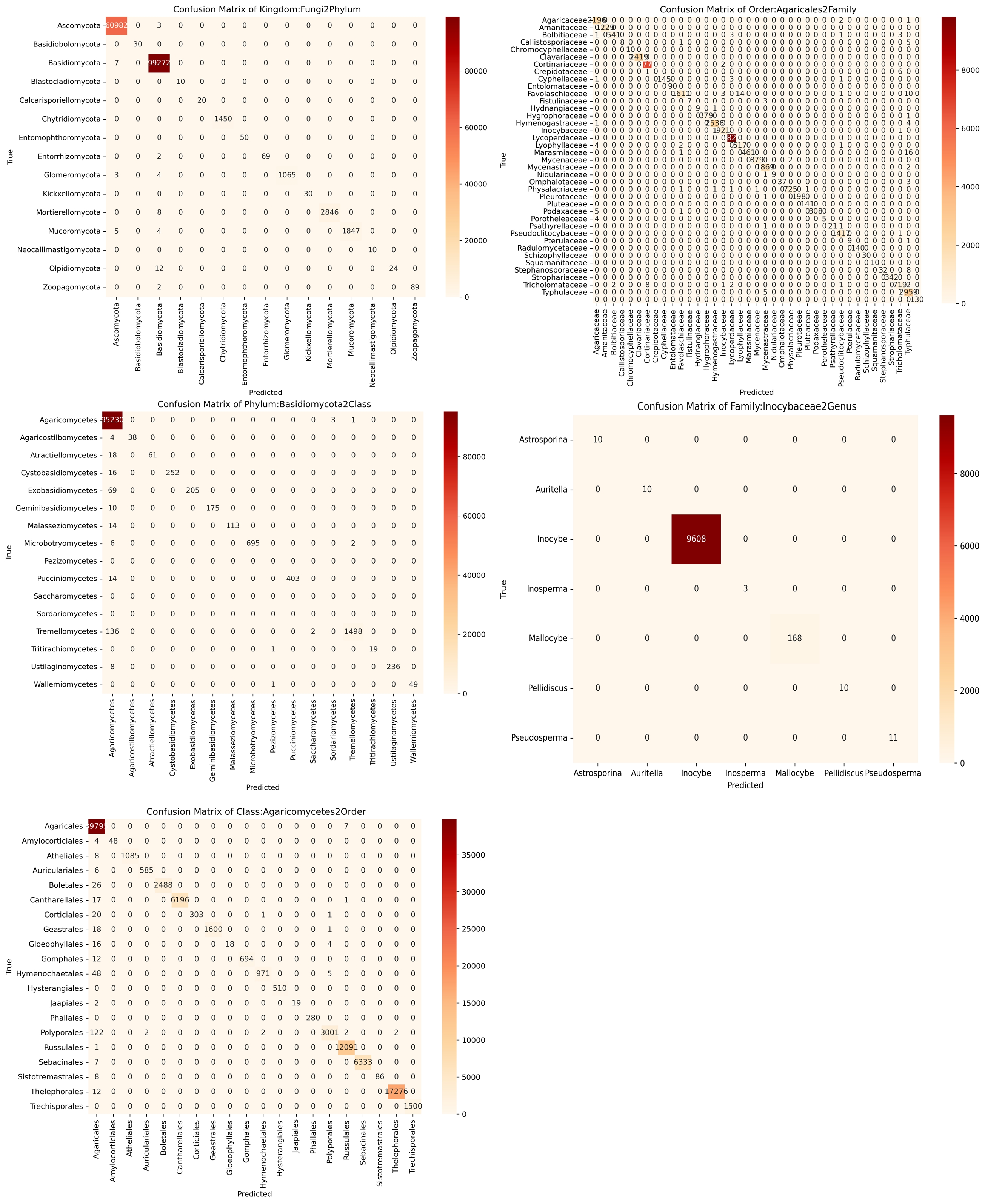

Supplement: Supplementary file 2 [file Image2.jpg]

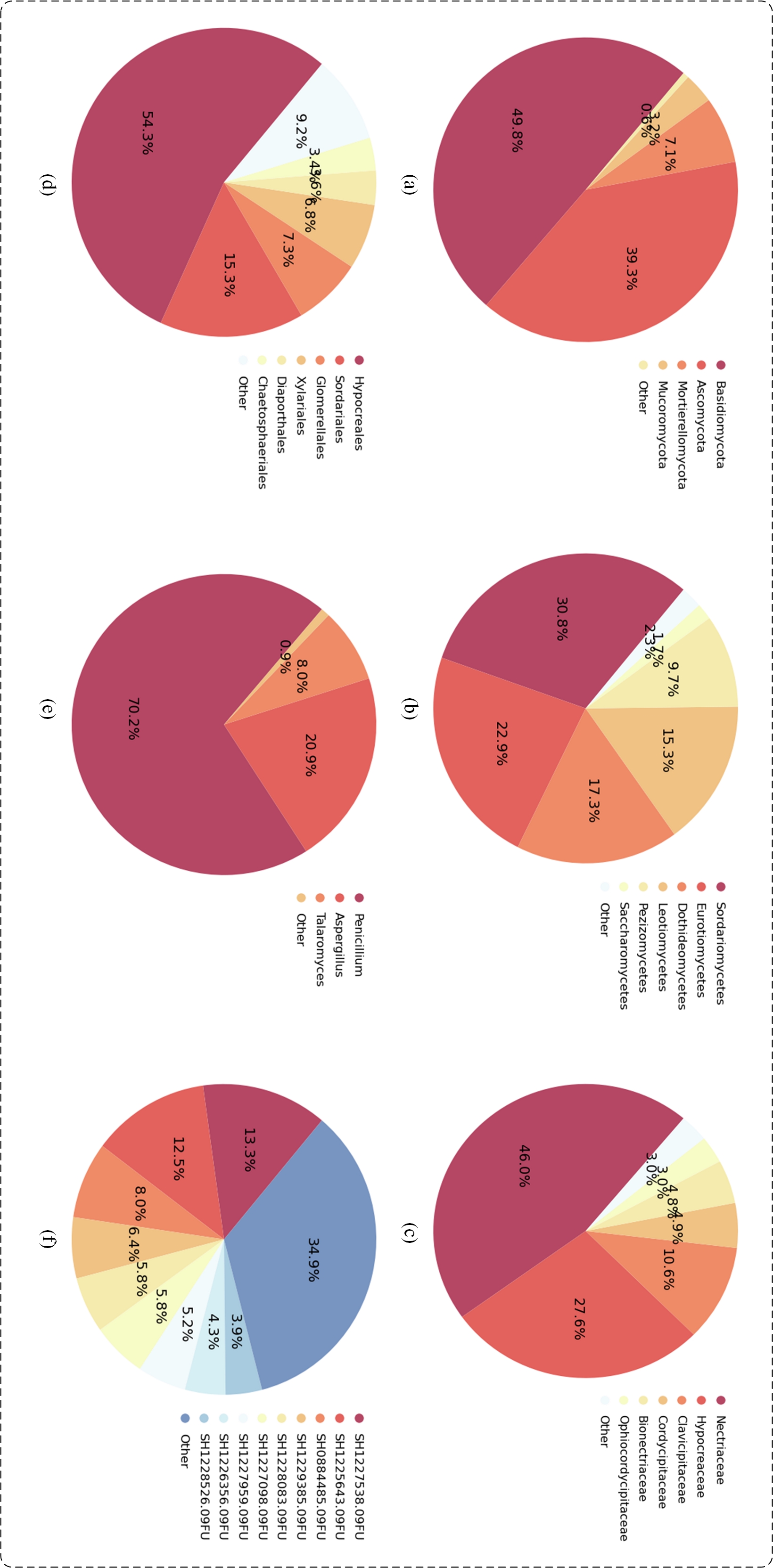

Supplement: Supplementary file 4 [file Image1.jpg]
